# A Bundled Approach to Pulmonary Tuberculosis Testing: Experience from a Tertiary Care Centre in South India

**DOI:** 10.7759/cureus.6042

**Published:** 2019-10-30

**Authors:** Balram Rathish, Arun Wilson, Roshni Pillay, Anup Warrier, Geetha Philips

**Affiliations:** 1 Internal Medicine, Aster Medcity, Kochi, IND; 2 Infectious Diseases, Aster Medcity, Kochi, IND

**Keywords:** tuberculosis bundle, mycobacterium culture, sputum microscopy, genexpert

## Abstract

The diagnosis of pulmonary tuberculosis (PTB) is based on a stepwise approach consisting of sputum microscopy and molecular testing (GeneXpert), with the use of Mycobacterium culture in select cases. We analysed a bundled approach of PTB testing, consisting of all three of these tests during the patient's first visit, for all patients fitting into the clinical criteria of PTB. We retrospectively analysed the medical records of all patients who underwent the TB diagnosis bundle during a period of 29 months. The sensitivity, specificity, positive predictive value (PPV) and negative predictive value (NPV) of sputum microscopy and GeneXpert were compared against the gold standard of Mycobacterium culture. The incremental yield of diagnosing PTB by addition of Mycobacterium culture in the diagnostic bundle during the initial testing was also calculated. The bundled approach conferred an advantage in terms of faster clinical decision and increased diagnosis rates, by virtue of the speed of smear microscopy and GeneXpert, combined with the higher sensitivity of culture. The bundle also had the additional benefit of detecting non-tuberculous Mycobacterium (NTM) by the culture method. Hence we feel that the bundled approach ensures fewer number of hospital visits and reduces the potential delays in a clinical decision making.

## Introduction

In India, Revised National Tuberculosis Control Program (RNTCP) recommends the use of sputum smear microscopy for acid fast bacilli (AFB), nucleic acid amplification test (GeneXpert), and if either of these tests are positive, then Mycobacterium culture for the diagnosis of pulmonary tuberculosis (PTB) [1]. Sputum microscopy is relatively inexpensive and quick, but has poor sensitivity as compared to the other two modalities [2]. GeneXpert provides accurate and rapid diagnosis, while culture is the most sensitive, but takes between two to eight weeks to yield results, and is not useful in rapid diagnostics [1].

In our centre, any patient suspected to have PTB, underwent a bundled PTB testing, with sputum or bronchoalveolar lavage (BAL) sample sent for AFB smear (both Ziehl-Neelsen as well as Auramine O staining with fluorescence microscopy), GeneXpert, and culture on both Middlebrook 7H9 broth readings as well as Löwenstein-Jensen solid medium.

Anti-tubercular treatment was given if any one of these tests were positive along with the high index of clinical suspicion.

## Materials and methods

We retrospectively analysed the medical records of all patients who had underwent the TB diagnosis bundle in the last 29 months between February 1, 2016 and June 30, 2018, who satisfied the inclusion criteria for presumptive pulmonary TB; all patients who had any of the symptoms and signs suggestive of PTB including cough of more than two weeks, fever of more than two weeks, significant weight loss, hemoptysis, any abnormality on chest radiograph [1].

The sensitivity, specificity, positive predictive value (PPV) and negative predictive value (NPV) of sputum microscopy and GeneXpert were compared against the gold standard of Mycobacterium culture. The incremental yield of adding Mycobacterium culture in the diagnostic bundle was also calculated.

## Results

A total of 1048 patients fitting into the clinical criteria for presumptive PTB underwent the bundled TB diagnostic testing at our centre in the last 29 months. One hundred and thirteen patients were bundle positive, i.e., had at least one test positive.

Eighty-eight patients were smear positive. Ninety-six patients were GeneXpert positive. Ninety patients were culture positive. The combined approach of smear plus GeneXpert yielded 105 patients, out of which 90 were also culture positive.

For sputum microscopy, the sensitivity was found to be 77.77%, specificity of 98.12%, PPV of 71.42% and NPV of 97.91%, compared against the gold standard of Mycobacterium culture (Table 1).

**Table 1:** Sensitivity, specificity, PPV and NPV of sputum microscopy. PPV: Positive predictive value; NPV: Negative predictive value.

SMEAR	TB	NO TB	TOTAL
SMEAR POSITIVE	70	18	88
SMEAR NEGATIVE	20	940	960
TOTAL	90	958	1048

For GeneXpert, the sensitivity was found to be 91.11%, specificity of 98.53%, PPV of 85.41% and NPV of 99.15%, compared against the gold standard of Mycobacterium culture (Table 2).

**Table 2:** Sensitivity, specificity, PPV and NPV of GeneXpert. PPV: Positive predictive value; NPV: Negative predictive value.

GENEXPERT	TB	NO TB	TOTAL
GENEXPERT POSITIVE	82	14	96
GENEXPERT NEGATIVE	8	944	952
TOTAL	90	958	1048

Eight patients were found to be only culture positive, with the other two tests being negative. The incremental yield of adding culture was found to be 14.6%.

Twenty-three out of the total 113 cases (20.35%) were either smear or GeneXpert positive, but culture negative. Four patients (4.16%) had Rifampicin resistant multi-drug resistant (MDR) TB. Three of these patients had previous exposure to first line ATT, while one patient was treatment naive.

In addition to the 113 PTB patients, 22 patients who were smear positive and GeneXpert negative, were further identified to have non-tuberculous Mycobacterium (NTM) by the culture method.

## Discussion

In India, the diagnosis of tuberculosis still remains a challenge due to the relatively low sensitivity and specificity of easily accessible tests like smear microscopy and the long turn around times of gold standard tests such as Mycobacterium culture. As per the national RNTCP guidelines, a patient who is clinically suspected to have tuberculosis first undergoes smear microscopy and Genexpert and if either of these tests are possible, further undergoes Mycobacterium culturing with drug susceptibility testing. The sensitivity of smear microscopy is relatively low even when three serial samples are analysed from the same patient [2]. For GeneXpert, results from 12 single centre evaluation studies with varying design and study population and reviewed by WHO reported the sensitivity in detecting TB from 70%-100% in culture positive patients and around 60% in those with smear negative disease and specificity ranging from 91%-100% and average rifampicin sensitivity and specificity around 98% and 99% [3, 4].

The relatively low sensitivity of smear leads to false negatives in numerous cases, especially in cases with a low bacterial load. The threshold for detection is high bacterial load for smear (>10,000 cfu/ml) and GeneXpert (130-150 cfu/ml) while that for culture is low (10-100 cfu/ml) [5-7]. Traditionally, solid culture media for mycobacteria are kept for up to eight weeks before a negative result is reported. Even though it is well documented that liquid media detect mycobacteria much earlier, most incubation protocols still require a maximum of six weeks [8]. The routine use of Mycobacterium culture is not preferred due to this long turn around time. GeneXpert, with its higher sensitivity and specificity, is a better tool with a rapid turn around time, but is still not as sensitive as Mycobacterium culture, which is considered the gold standard test [9].

In our study, the sensitivity, specificity, PPV and NPV of smear microscopy was higher than previously reported studies, while that of GeneXpert was similar to previously reported studies [2-4]. Our particular point of interest was the added yield of diagnosis that was obtained by the addition of the third test, i.e., Mycobacterium culture at the first visit, rather than depending on positivity of either smear microscopy or GeneXpert, and this was found to be 14.6%.

The bundled approach hence conferred an advantage in terms of faster clinical decision and increased diagnosis rates, by virtue of the speed of smear microscopy and GeneXpert, combined with the higher sensitivity of culture. In addition to the increased diagnostic yield, the added advantage that addition of Mycobacterium culture into the diagnostic bundle conferred was the detection of an additional 22 cases of NTM. This was due to the inability of GeneXpert to detect NTM and the relatively low sensitivity of smear microscopy. These cases would have gone undiagnosed if not for culture, and the patients would not have been treated on time. The bundle also ensures fewer number of hospital visits and reduces the potential delays in a clinical decision making.

## Conclusions

We recommend a combined approach of smear microscopy with concurrent GeneXpert and Mycobacterium culture for diagnosis of PTB during the initial testing. In our analysis, a bundled approach showed an increased pick up rate for Tuberculosis bacilli in the pulmonary samples and additional pick up of NTM which would have been missed if not for Mycobacterium culture. The smear microscopy gives the advantage of being relatively inexpensive with a fast turn around time, but has comparatively lower sensitivity and specificity. The GeneXpert also provides a rapid turn around time with higher sensitivity and specificity, along with identification of Rifampicin resistance, which may help in direction of treatment regimens. The Mycobacterium culture can pick up cases with very low bacterial load and confers an incremental yield with identification of NTM which may be clinically relevant. Hence, a combination of these three tests at the outset for a patient suspected to have pulmonary tuberculosis may virtually diagnose or exclude pulmonary tuberculosis without any ambiguity. Further similar studies may be warranted to reproduce similar results in other centres, which may add strength to this approach to diagnosis of PTB.

## References

[1] India India, M. (2018 (2019). TOG-Chapter 3-Case finding & diagnosis strategy: Ministry of Health and Family Welfare. [online] Tbcindia.gov.in. https://tbcindia.gov.in/index1.php?lang=1&level=2&sublinkid=4573&lid=3177.

[2] Mathew P, Kuo YH, Vazirani B, Eng RH, Weinstein MP (2002). Are three sputum acid-fast bacillus smears necessary for discontinuing tuberculosis isolation?. J Clin Microbiol.

[3] Zeka AN, Tasbakan S, Cavusoglu C (2011). Evaluation of the GeneXpert MTB/RIF assay for rapid diagnosis of tuberculosis and detection of rifampin resistance in pulmonary and extrapulmonary specimens. J Clin Microbiol.

[4] Agrawal M, Bajaj A, Bhatia V, Dutt S (2016). Comparative study of GeneXpert with ZN stain and culture in samples of suspected pulmonary tuberculosis. J Clin Diagnostic Res.

[5] Sahana KS, Prabhu AS, Saldanha PR (2018). Usage of cartridge based nucleic acid amplification test (CB-NAAT/GeneXpert) test as diagnostic modality for pediatric tuberculosis; case series from Mangalore, South India. J Clin Tuberc Other Mycobact Dis.

[6] Groothuis DG, Yates MD (1991). Decontamination, microscopy and isolation. Diagnostic and Public Health Mycobacteriology.

[7] Garcia LS, Isenberg HD (2010). Clinical Microbiology Procedures Handbook. American Society for Microbiology Press;.

[8] Pfyffer GE, Wittwer F (2012). Incubation time of mycobacterial cultures: how long is long enough to issue a final negative report to the clinician?. J Clin Microbiol.

[9] Forbes BA, Hall GS, Miller MB (2018). Practice guidelines for clinical microbiology laboratories: mycobacteria. Clin Microbiol Rev.

